# Inorganic-based biomaterials for rapid hemostasis and wound healing

**DOI:** 10.1039/d2sc04962g

**Published:** 2022-11-30

**Authors:** Yi Zheng, Jinfu Wu, Yufang Zhu, Chengtie Wu

**Affiliations:** a State Key Laboratory of High Performance Ceramics and Superfine Microstructure, Shanghai Institute of Ceramics, Chinese Academy of Sciences No. 1295 Dingxi Road Shanghai 200050 People's Republic of China zhuyufang@mail.sic.ac.cn chengtiewu@mail.sic.ac.cn; b Center of Materials Science and Optoelectronics Engineering, University of Chinese Academy of Sciences No. 19(A) Yuquan Road Beijing 100049 People's Republic of China

## Abstract

The challenge for the treatment of severe traumas poses an urgent clinical need for the development of biomaterials to achieve rapid hemostasis and wound healing. In the past few decades, active inorganic components and their derived composites have become potential clinical products owing to their excellent performances in the process of hemorrhage control and tissue repair. In this review, we provide a current overview of the development of inorganic-based biomaterials used for hemostasis and wound healing. We highlight the methods and strategies for the design of inorganic-based biomaterials, including 3D printing, freeze-drying, electrospinning and vacuum filtration. Importantly, inorganic-based biomaterials for rapid hemostasis and wound healing are presented, and we divide them into several categories according to different chemistry and forms and further discuss their properties, therapeutic mechanisms and applications. Finally, the conclusions and future prospects are suggested for the development of novel inorganic-based biomaterials in the field of rapid hemostasis and wound healing.

## Abstract 2

This review provides the recent progress in the application of inorganic-based biomaterials for rapid hemostasis and wound healing, including preparation methods, material systems, intrinsic mechanisms and biofunctions.

## Graphical abstract

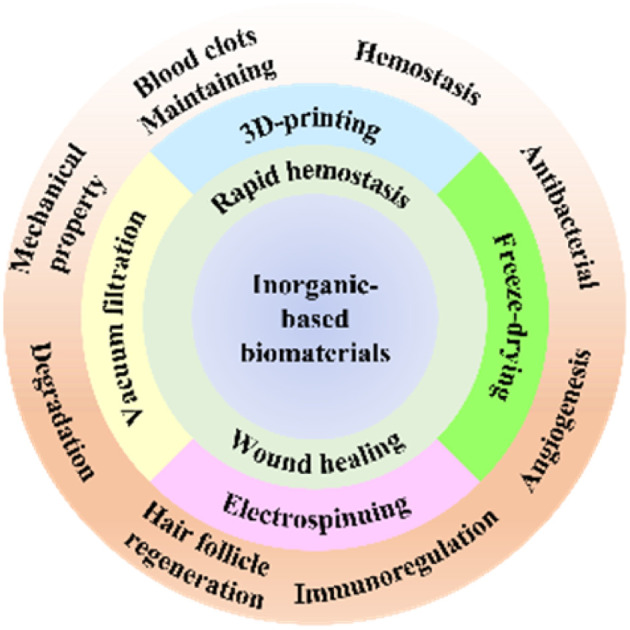

## 1. Introduction

Emergencies are still the number one reason for premature death worldwide; therefore, rapid hemorrhage control and effective wound healing are of great importance in medical care.^1–3^ Bleeding caused by minor wounds is usually stopped by intrinsic hemostatic mechanisms, and minor wounds can also be self-healed by the body. However, uncontrolled hemorrhage and healing difficulties following severe trauma are associated with high mortality.^4,5^ Rapid control of bleeding and promotion of wound healing are imperative and have motivated the development of hemostatic biomaterials and wound healing biomaterials. Hemostatic biomaterials with excellent absorption capacity and procoagulant ability can rapidly aggregate blood cells and platelets and accelerate the conversion of fibrinogen to fibrin in blood, thereby forming a physical barrier to prevent further bleeding.^6^ Wound healing biomaterials contribute to functions including antibacterial, immunomodulatory, fluid absorption, maintaining a moist environment, *etc.*, which promote cell proliferation, angiogenesis and tissue regeneration, leading to a great reduction of the healing time.^7^ At present, the application of biomaterials in hemostasis and wound healing is mostly separated in clinical cases, and this results in a certain amount of inconvenience and waste. Meanwhile, more and more reports show that there is a tight connection between hemostasis and wound healing, which inspires researchers to prepare biomaterials for both rapid hemostasis and wound healing.^8–13^ Ideal biomaterials for hemostasis and wound healing should rapidly control bleeding, be easy to apply to different types of complex wounds, be biocompatible and non-cytotoxic, be good for wound healing and be easy to remove or biodegrade. Besides, the preparation process should be simple and efficient, and long-term stability during storage can be achieved.

The two main factors that need to be considered are the active chemical components and material forms when developing biomaterials for hemostasis and wound healing. Active chemical components can be divided into organic components and inorganic components according to chemical composition. Active organic components include natural polymers such as chitosan,^14,15^ alginic acid,^16^ gelatin,^17^ and synthetic polymers such as polyvinyl alcohol^18^ and GelMA.^19^ Active inorganic components include natural minerals,^20^ synthetic silicates,^21^ phosphates,^22^*etc.* Compared with organic biomaterials, inorganic biomaterials with unique advantages or characteristics show better potential in hemostasis and wound healing. On the one hand, inorganics generally exhibit much higher surface energy than organics, which makes them hydrophilic and even super-hydrophilic. Subsequently, inorganic biomaterials can quickly absorb water and achieve ultra-rapid hemostasis. On the other hand, inorganic biomaterials possess various functions like bioactive ion release, photothermal properties, magnetothermal properties, photodynamic properties or conductivity, which endow them with good prospects for future development. Hence, we mainly focus on exploring inorganic-based biomaterials for rapid hemostasis and wound healing in this review. Active inorganic components show the advantages of good biocompatibility, high bioactivity, absorption of blood, promotion of blood coagulation and release of bioactive ions, which have attracted the interest of researchers.^7^ However, active inorganic components are mostly micro–nano-scale particles whose applications are limited. Preparing inorganic-based biomaterials with more useful characteristics by combining novel methods, strategies and manufacturing techniques still remains a huge challenge. Many forms of inorganic-based biomaterials have been developed, ranging from simple to complex, from 2D to 3D, from dense to porous, from hydrophilic to hydrophobic and from macrosized to nanosized. Importantly, the manufacturing technique is the key to determining the material form that influences the functions and applications of an inorganic-based biomaterial. Representative manufacturing techniques include 3D printing,^23–25^ freeze-drying,^26–28^ electrospinning^29,30^ and vacuum filtration,^31,32^ which endow inorganic-based biomaterials with the forms of 3D scaffolds, porous bulk, soft membranes or tough hydrogels. The diverse forms of inorganic-based biomaterials are suitable for various and complex wounds—no matter hard or soft tissue wounds, or even superficial, deep, and irregular wounds. Hence, it is important to decide on appropriate active inorganic components and manufacturing techniques.

**Fig. 1:**
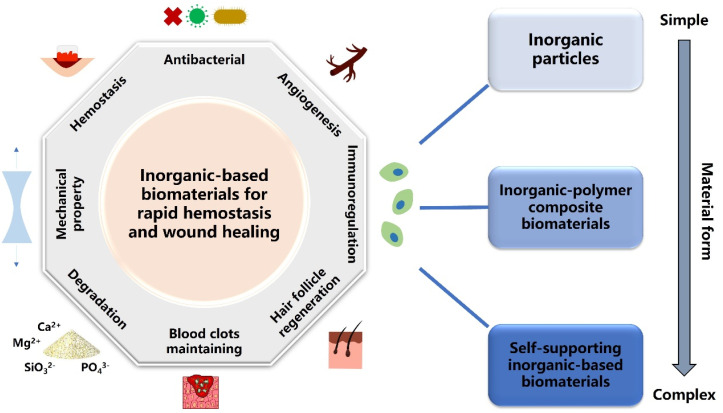
Schematic illustration of the three types of inorganic-based biomaterials classified by the complexity of the material form for various applications of rapid hemostasis and wound healing.

Generally, inorganic-based biomaterials for rapid hemostasis and wound healing are divided into three categories—inorganic particles, inorganic–polymer composite biomaterials and self-supporting inorganic-based biomaterials. Moreover, different material forms and active inorganic components endow these biomaterials with various properties, such as hemostasis, antibacterial activity, angiogenesis and degradation (Fig. 1). For hemostasis, natural mineral clays act as a molecular sieve to absorb water, thereby concentrating various clotting factors, platelets and blood cells over the site of the wound to form a plug.^33^ However, the exothermic reaction upon water absorption leads to thermal damage to surrounding tissue and even necrosis, severely limiting the application of natural mineral clays.^34^ Therefore, researchers have prepared silica-based mesoporous particles, such as mesoporous silica and bioactive glasses, which display good hemostatic properties and biocompatibility.^35^ Furthermore, various inorganic–polymer composite hemostatic materials and self-supporting inorganic-based hemostatic materials such as sponges,^36,37^ aerogels,^38^ hydrogels,^39^ and membranes^40^ have been developed to meet the needs of different clinical applications. These novel inorganic-based hemostatic biomaterials show inspiring development prospects because they have better hemostatic activity and can be applied to deep and complex wounds. As for wound healing, biomaterials with forms of basic dressings or scaffolds play the role of fundamental fixing and supporting during the wound healing process.^41^ On the other hand, multifunctional inorganic-based wound healing biomaterials have been developed to induce specific therapeutic effects on wound healing. These multifunctional biomaterials possess antibacterial, photothermal, immunoregulation, angiogenesis, and hair follicle regeneration functions, which can synergistically accelerate wound healing.^42^ Recently, intelligent responsive inorganic-based biomaterials have attracted attention because of their great importance in extending therapeutic methods of wound healing. Intelligent response means that the materials can respond to physical stimuli such as electricity, light, pH and temperature.^43,44^ It helps patients to monitor the wound healing process in time or regulate the appropriate healing microenvironment, so that biomaterials can promote wound healing more effectively.

Many studies demonstrate a strong link between hemostasis and wound healing. In brief, the blood clots formed during hemostasis play an essential role in the subsequent wound healing process.^45–47^ Blood clots are composed mainly of platelets and fibrin, and they are a viable, dynamic matrix of proteins and cells, which not only contributes to hemostasis but also serves as a provisional lattice for incoming inflammatory cells, fibroblasts and growth factors.^48^ Moreover, blood clots could also regulate the immune microenvironment at the wound site during the process of formation and fibrinolysis.^47^ Therefore, if biomaterials could stop bleeding rapidly and be removed without causing secondary damage or be retained at the wound site for degradation, the remaining blood clots will further promote wound healing and achieve a better integrated treatment effect. To date, a number of inorganic-based biomaterials for rapid hemostasis and wound healing have been prepared, mainly including surface-modified natural mineral clays and inorganic–polymer composite sponges, which hold the abilities of excellent hemostatic performances, biocompatibility, and bioactive ion release behavior to promote wound healing.^49–52^ Meanwhile, scientists are developing inorganic-based biomaterials with multifunctional synergistic effects such as hemostasis, antibacterial, immunoregulation, photothermal therapy and wound healing. Therefore, using only one material to meet the therapeutic needs of complex wounds represents the promising development future of inorganic-based biomaterials for rapid hemostasis and wound healing.^30,53–55^ Nevertheless, there are several challenges of inorganic-based biomaterials for hemostasis and wound healing. Firstly, more and more active inorganic components with better effects of hemostasis and wound healing should be developed to address more complex clinical situations, such as the need for ultra-rapid hemostasis in the battlefield and skin regeneration of extensive cutaneous burns. Secondly, it is critical to fabricate active inorganic components in bulk forms instead of powder forms for extensive clinical application. Thirdly, the molecular mechanisms of inorganic-based biomaterials for rapid hemostasis and wound healing still need to be further investigated and explored.

In this review, our aim is to summarize the recent progress on inorganic-based biomaterials for rapid hemostasis and wound healing, especially focusing on the chemical components, preparation methods and strategies, and functional roles of hemostasis and wound healing. We provide an overview and discuss the development of chemical modification and manufacturing techniques over the past 10 years. Moreover, we describe the different forms of inorganic-based hemostatic biomaterials and wound healing biomaterials, highlighting their advantages, disadvantages and applications. Additionally, the designs, developments and challenges of novel inorganic-based biomaterials for both rapid hemostasis and wound healing and multifunctional synergetic inorganic-based biomaterials are also discussed to inspire more in-depth research in this area.

## 2. Methods and strategies for the design of novel inorganic-based biomaterials

Clearly, material design is the key process that determines the structures, properties and performances of materials, which has inspired plenty of researchers to explore various methods and strategies for preparing inorganic-based biomaterials. As known, a variety of active inorganic components ranging from nano- to micro-scales have been synthesized by chemical methods such as hydrothermal, sol–gel, solid reaction, precursor-derived method, *etc.*^56–59^ Nevertheless, it is difficult to directly apply these active inorganic components in clinics. Therefore, preparing useable inorganic-based biomaterials by utilizing suitable manufacturing techniques is extremely essential. In this section, we provide an overview of several common manufacturing techniques, and subsequently focus on how these manufacturing techniques determine the forms, properties and applications of inorganic-based biomaterials.

### 2.1. 3D printing

3D printing has become a critical technique to manufacture clinical biomaterials.^60^ As known, 3D printing is to develop materials with a three-dimensional structure through layer-by-layer stacking. Conventionally, the 3D printing process can be simply described as follows: first, a 3D model is established with computer-aided design (CAD) software, and then the established 3D model is printed along a preset path through a robotic arm, a nozzle or other apparatus.^61^ Nowadays, the 3D printing technique has also been widely applied in fabricating biomaterials for hemostasis and wound healing. The biomaterials prepared by the 3D printing possess reliable mechanical strength, good biocompatibility and a connected macroporous structure. Hence, they can play a role of support and fixation at the wound site and further provide channels for cell migration and nutrient delivery.

#### 2.1.1. Extrusion-based 3D printing

Extrusion-based 3D printing is the earliest developed technology of 3D printing. Under the preset CAD program, the nozzle-loaded ink is printed and stacked layer by layer to obtain a three-dimensional scaffold. For inorganic-based 3D printed scaffolds, high temperature treatment could remove the organic binder and densify the particles, thereby greatly improving their mechanical strength.^62,63^ For instance, a 3D scaffold dressing composed of decellularized small intestinal submucosa, mesoporous bioactive glass and exosome was fabricated by Hu *et al.* The introduction of mesoporous bioactive glass increased the printability of the slurry and the bioactivity of the scaffolds. Interestingly, this scaffold dressing could accelerate diabetic wound healing through increasing the blood flow of the wound and stimulating the angiogenesis process.^64^ For a long time, our group have 3D printed a series of inorganic-based scaffolds based on bioceramics and bioglasses. These scaffolds have cross-connected porous structures of hundreds of microns that provide an environment for nutrient delivery and cell migration. Furthermore, the released bioactive ions from these scaffolds can also promote cell proliferation and differentiation.^65–68^ In another study, Li *et al.* fabricated a dopamine-modified bioceramic scaffold of which the mussel-inspired surface could stimulate the paracrine effect of adipose-derived MSCs. Interestingly, such a bioceramic scaffold showed abilities to accelerate wound closure and enhance vascularization.^69^

#### 2.1.2. Digital laser processing-based 3D printing

Digital laser processing-based 3D printing is a manufacturing technique based on a light-activated polymerization reaction, in which polymers are polymerized through selective laser irradiation to form a three-dimensional structure.^70^ Combining bioceramics with polymers, scientists can fabricate inorganic-based 3D printed scaffolds with a complex and precise internal structure through digital laser processing-based 3D printing.^71^ For instance, Sakurai *et al.* investigated the principal components of hemostasis based on a microengineered vascularized bleeding model. Contributing to the excellent rheological properties and formability of polydimethylsiloxane (PDMS), a bleeding model consisting of three PDMS layers that mimic the structure of a natural blood vessel had been successfully fabricated. The experimental results further showed the essential functions of platelets, von Willebrand factor and endothelial phosphatidylserine in hemostasis. This biomimetic bleeding model held significant promise as a therapeutic-guiding tool for hemostasis.^72^ In another study, Kim and co-workers developed a methacrylated photocurable silk fibroin bioink with excellent printability, mechanical and rheological properties, and biocompatibility, which could be used broadly including in nerve tissue engineering, trachea tissue engineering and wound healing.^73^ Inspired by the mechanical properties of natural blood vessels, Bracaglia *et al.* designed a printable and degradable hybrid scaffold. The experimental results showed that the phenotype of macrophages could be affected by changing the components of this scaffold, thereby regulating inflammation and promoting wound healing.^74^

#### 2.1.3. 3D bioprinting

With the development of tissue engineering, the traditional “inanimate” 3D printing scaffolds are difficult to meet the complex clinical needs. Therefore, 3D bioprinting has been proposed and flourished. The most significant feature of 3D bioprinting is the use of “bioink” that contains living cells. Through 3D bioprinting, cells can be pre-grown, differentiated *in vitro*, and then implanted into the body, which greatly improves the bioactivity and therapeutic effect of the materials.^75^ For example, Biranje *et al.* designed a cellulose nanofibril and casein composite bioink that has a viscoelastic behavior, stability, homogeneity and better printability. Furthermore, the 3D printed cell-laden composite scaffold could promote the growth and proliferation of NIH 3T3 fibroblast cells.^76^ In another study, Turner *et al.* reported a novel double-peptide functionalized GelMA bioink and directly deposited BMSCs and HUVECs to prepare a core/shell bioprinting scaffold. Encouragingly, this core/shell scaffold showed good physico/chemical characteristics that supported the propagation and early development of vascular cells in a tube-like structure.^77^ Recently, our group reported a Li–Mg–Si bioceramic-GelMA hydrogel composite bioink, which was utilized to construct a co-culture scaffold containing bone marrow mesenchymal stem cells (hpMSCs) and chondrocytes (RCs). Owing to the introduction of Li–Mg–Si bioceramic, the printability of bioink was improved and the bioactivity of the scaffold was also increased. *In vitro* and *in vivo* experiments have verified that the scaffold containing Li–Mg–Si bioceramic exhibited the function of stimulating multiple cells for differentiation towards specific directions, thereby accelerating the repair and regeneration of severe osteochondral defects.^78^

### 2.2. Freeze-drying

Freeze-drying, also known as ice templating, is a particularly versatile technique. It has been applied extensively for the fabrication of well-controlled biomimetic porous materials based on ceramics, metals, polymers, biomacromolecules and carbon nanomaterials.^79^ Briefly, freeze-drying involves the controlled solidification of a solution, suspension, sol or gel, followed by the sublimation of the solvent under reduced pressure.^80,81^ Moreover, one outstanding advantage of freeze-drying is that the micro/macro-structures of the prepared freeze-drying scaffolds can be easily tailored by adjusting the process parameters.^82^ Therefore, biomaterials manufactured by freeze-drying have been developed into the most common forms for rapid hemostasis and wound healing benefitting from their porous structure and absorption capacity.

#### 2.2.1. Unidirectional freeze-drying

In the case of conventional unidirectional freezing-drying, the suspension starts to freeze under a single temperature gradient, causing the nucleation of ice to occur randomly on the cold surface and grow radially. As a result, the solidified suspension media consist commonly of microscale crystallites oriented preferentially along the direction of freezing, and constructing scaffolds with cellular porous structures.^79^ For instance, Yuan *et al.* developed a novel hemostatic nanocomposite fabricated by coupling oxidized bacterial cellulose and chitosan with collagen. These three raw materials possessed both good formability and excellent hemostatic properties, and excellent hemostatic efficacy of the composite was confirmed *in vivo*.^83^ Huang *et al.* prepared a series of biodegradable interpenetrating polymer network (IPN) dry cryogel hemostats. Owing to the IPN structure, the hemostat showed good injectability, robust mechanical and shape memory properties.^84^ Similarly, a series of antibacterial and antioxidant tissue-adhesive cryogels based on chitosan and polydopamine were designed by Li and co-workers, and these cryogels possessed good platelet adhesion, enrichment and activation properties.^85^ In another study, Lan *et al.* fabricated a chitosan/gelatin/sodium hyaluronate hemostatic dressing with silver nanoparticles. Additionally, this dressing had high blood absorption and promoted platelet aggregation by the activation of a positively charged surface and stimulation of silver nanoparticles.^86^

#### 2.2.2. Bidirectional freeze-drying

Apart from unidirectional freeze-drying, bidirectional freeze-drying is another important manufacturing technique in the preparation of biomaterials with “brick and mortar” (BM) structures.^87^ In bidirectional freezing, solid phase building blocks assemble into a large-size single-domain aligned lamellar structure, which is attributed to the controlled nucleation of ice crystals that allow the ice to grow both vertically and horizontally.^88,89^ From this perspective, active inorganic components with the morphology of nanosheets or nanowires are more likely to be fabricated into biomaterials with lamellar structures through bidirectional freeze-drying. For example, Bai and co-workers first reported on a bidirectional freezing technique to successfully assemble ceramic particles into a scaffold with a large-scale aligned, lamellar, porous, nacre-like structure and long-range order on the centimeter scale. Encouragingly, the scaffold possessed extremely high compressive strength.^90^ In a similar case, Li *et al.* of our group utilized akermanite and the bidirectional freeze-drying technique to construct hot dog-like biomaterials and “brick-and-mortar” structural biomaterials. These scaffolds displayed excellent properties including the high loading and sustained release of drugs and proteins and the improvement of the migration and differentiation of tissue cells.^91,92^ In addition to the above, Feng *et al.* reported a bio-inspired lamellar chitosan scaffold with a long range ordered porous structure. Most interestingly, the chitosan scaffold was found to be capable of inducing macrophage differentiation to the M2 phenotype that played an important role in tissue regeneration.^93^

### 2.3. Electrospinning

Electrospinning is the most widely developed manufacturing technique for preparing soft fiber membrane materials. Conventionally, a typical apparatus for electrospinning is cost-effective and straight forward. It consists of a high voltage supplier, a capillary tube with a small diameter needle or pipet, and a grounded fiber collecting screen.^94,95^ As is known, inorganic-based bio-membranes can be prepared by mixing bioceramic powders and polymers, while the physicochemical properties can be flexibly adjusted. Importantly, inorganic-based bio-membranes possessing excellent flexibility can be wrapped and applied to wounds like bandages. Furthermore, they show the multiple functions of hemostasis, bioactive ion release and wound healing promotion, thereby having great potential in tissue engineering.

Electrospinning bio-membranes have excellent flexibility, mechanical strength and absorption capacity, which can be easily used in hemostasis, healing and therapy of soft tissue wounds. Hence, a series of studies have illustrated their applications in hemorrhage control. For instance, Li *et al.* prepared a curcumin-loaded mesoporous silica incorporated nanofiber mat. The mesoporous silica with an extremely high surface area exhibited excellent hemostatic activity. Interestingly, the hybrid nanofiber mat could rapidly transform into a hydrogel when contacted with blood, and then activate the clotting system to stop bleeding.^97^ Long and co-workers described an emerging bifunctional hybrid fiber membrane incorporated with ZnO–Fe_2_O_3_ and kaolinite nanoclay for the first time, and the results showed that ZnO–Fe_2_O_3_ and kaolin possessed hemostatic, antibacterial and anti-inflammatory activities for favorable therapeutic efficacy.^98^

**Fig. 2:**
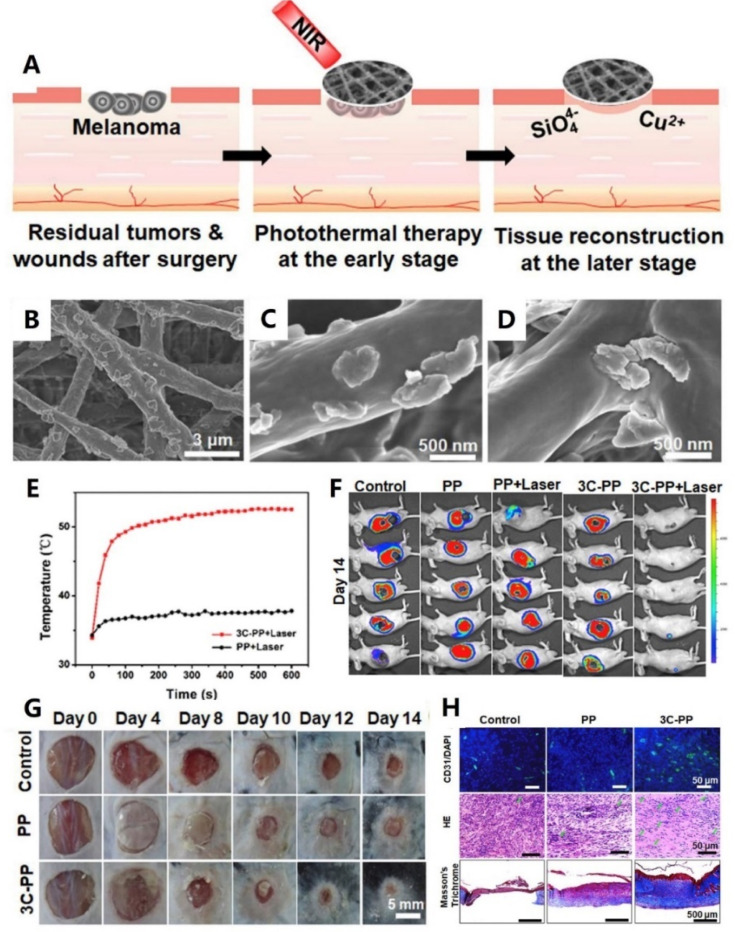
(A) Schematic illustration of the CaCuSi_4_O_10_ nano-sized particle (NP)-coated fibrous scaffolds for photothermal therapy and tissue regeneration. (B–D) SEM images of the CaCuSi_4_O_10_ NP-coated fibrous scaffolds. (E) Temperature changes of the tumors treated with laser-irradiated PP and 3C-PP scaffolds (0.45 W cm^−2^, 15 min). (F) The whole-body fluorescence imaging of tumors on day 14 (*n* = 5). (G) Wound photographs at chronic wounds of different groups after fibrous scaffold implantation. (H) Immunofluorescence (green: CD31 and blue: nuclei), H&E staining and Masson's Trichrome staining images of skin wounds on day 14. Adapted with permission from ref. 96. Copyright 2018, Elsevier Ltd.

In addition to hemostasis, here are their extensive developments and applications in skin or other soft tissue wound healing. Inspired by the design strategy and fabrication method of Chinese sesame sticks, our group developed a scaffold through spin coating of CaCuSi_4_O_10_ nanoparticles on the surface of electrospinning fibers (As shown in Fig. 2). Notably, the CaCuSi_4_O_10_ nanoparticles not only improved the stability of this fiber scaffold, but also endowed the scaffold with high bioactivity. *In vivo* evaluation proved that the scaffold could release bioactive Cu^2+^ and SiO_4_^4−^ to promote chronic wound healing.^96^ In another study, Chen and co-workers prepared a nanobioglass incorporated chitosan-PVA tri-layer nanofibrous membrane. Interestingly, the spatially designed structure optimized the functions of each component. *In vivo* experiments further showed that this membrane could significantly accelerate wound healing.^99^ Additionally, Balakrishnan *et al.* reported an Al_2_O_3_-based PVP nanofibrous scaffold. Contributing to the incorporation of Al_2_O_3_, the composite nanofibrous scaffold not only possessed improved porosity and degradation but also obtained a sustained release of Al_2_O_3_ nanoparticles to promote cell proliferation.^100^

### 2.4. Vacuum filtration and evaporation-induced self-assembly

Vacuum filtration and evaporation-induced self-assembly are also two typical manufacturing techniques to fabricate membrane materials. As is known, these two methods directly utilize building blocks and perform a simple bottom-up and layer-by-layer stacking strategy to fabricate inorganic-based bio-membrane materials.^101^ Besides, the physicochemical properties of the membranes can be easily controlled by adjusting the concentration of the building block suspension. Consequently, bio-membrane materials with a high inorganic content can be prepared by vacuum filtration or evaporation-induced self-assembly techniques. These bio-membrane materials show great potential in clinical treatment due to their excellent mechanical properties and bioactivities.

Numerous studies have presented inorganic-based bio-membranes with favorable performances by using vacuum filtration or evaporation-induced self-assembly. For instance, Mansoorianfar *et al.* prepared densely packed cellulose layers impregnated with 58S bioglass nanoparticles. Interestingly, the effective integration of bioglass nanoparticles between cellulose interlayers increased the hemostatic activity of the developed fabric by 50%.^102^ Inspired by the close relationship between the strength and hierarchical structure of nacre, Xue *et al.* fabricated a hierarchical and porous graphene oxide–chitosan–calcium silicate membrane biomaterial, in which graphene oxide was the main component, chitosan acted as a binder and calcium silicate provided bioactivity. Moreover, this bioinspired hierarchical material possessed high tensile strength, compatible breathability, water absorption and ideal photothermal performance, which significantly promoted wound healing.^103^ For evaporation-induced self-assembly, Yu and co-workers demonstrated that a large-sized, three-dimensional bulk artificial nacre could be facilely fabricated *via* a bottom-up assembly process. Furthermore, this artificial nacre comprehensively mimicked the hierarchical structures and toughening mechanisms of natural nacre based on laminating pre-fabricated two-dimensional nacre-mimetic membranes. Obviously, such three-dimensional bulk artificial nacre would be promising for hemostasis and wound healing owing to its porous and hierarchical structure.^104,105^

## 3. Inorganic-based biomaterials for rapid hemostasis

### 3.1. Hemostasis mechanisms

**Fig. 3:**
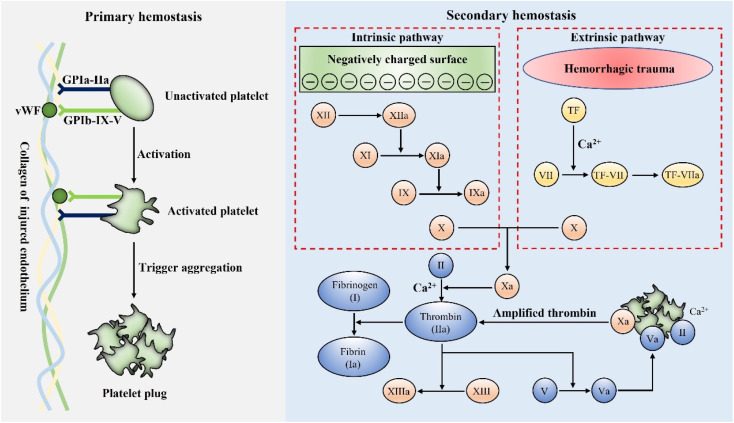
Schematic illustration of the mechanisms of primary hemostasis and secondary hemostasis.

Hemostasis, or coagulation, is a naturally occurring process that results in forming a stable and insoluble blood clot at an injury site to prevent further bleeding.^106^ The formation of blood clots is activated within a few seconds of injury and localized only at the wound site.^107^ Therefore, coagulation is a very accurate process to avoid the risk of thromboembolism.^108^ Hemostasis primarily consists of two stages, primary and secondary hemostasis. Fig. 3 schematically presents the details of the hemostasis process.

Generally, the primary hemostasis occurs immediately when bleeding occurs. In the process of primary hemostasis, platelets quickly adhere to the injured endothelium and are activated through specific receptors, namely GPIa-IIa and GPIb-IX-V.^109,110^ The activated platelets then trigger the aggregation and formation of a platelet plug to stop bleeding.^111,112^ However, the platelet plug is fragile and the secondary hemostasis is essential to further form a stable blood clot. In the process of secondary hemostasis, coagulation factors undergo a complex coagulation cascade, which includes extrinsic and intrinsic pathways. The extrinsic pathway is initiated by blood exposure to tissue factor (TF) and eventually leads to the activation of factor Xa, while the intrinsic pathway is initiated when blood contacts a negatively charged surface, which also results in the activation of factor Xa. Subsequently, the common factor Xa can produce a large amount of prothrombin (II) into thrombin (IIa) under the synergetic effect of phospholipids and Ca^2+^.^113^ As is known, thrombin is the key factor, which can convert fibrinogen (I) into fibrin (Ia), and the interwoven fibrin entangles platelets and blood cells to form a firm and stable blood clot at the bleeding site, which can stop bleeding more effectively.^109,111^

For hemostatic materials, it is possible to accelerate hemostasis by regulating a certain process in the complex coagulation reaction through specific properties. As an example, anionic polysaccharides represented by chitosan possess a positively charged group –NH^3+^, which can accelerate coagulation by aggregating and activating platelets in hemostasis.^114,115^ Besides, natural mineral clays such as zeolites and kaolin exhibit a mesoporous structure and Ca^2+^ release ability to promote hemostasis.^33^ In the following section, we focus on four major types of inorganic-based hemostatic materials, further discussing their properties and hemostatic mechanisms.

### 3.2. Natural mineral clays

As is known, natural mineral clays are composed of unreactive oxides of magnesium, aluminum, sodium, silicon, and minute amounts of quartz. These agents can rapidly absorb water to gather platelets, blood cells and coagulation factors at the injury site. They can also activate the secondary hemostasis, thereby accelerating blood clotting.^33^ Zeolites, kaolin, and montmorillonite are the three most widely used natural mineral clay hemostats. Furthermore, a variety of inorganic-based hemostatic materials based on natural mineral clays have been approved for battlefield trauma, field first aid and clinical hemostasis.

#### 3.2.1. Zeolites

Zeolites, microporous crystalline aluminosilicate minerals found in nature, are composed of a tetrahedral SiO_2_ framework to form a three-dimensional structure.^116^ When in contact with blood, zeolites can absorb a large volume of water to gather platelets, blood cells and coagulation factors. Meanwhile, the released Ca^2+^ from zeolites activates the intrinsic pathway, further accelerating coagulation. In a recent study, Shang *et al.* revealed the hemostatic mechanism of Ca-zeolites at the molecular level.^117^ A prothrombin-to-thrombin conversion on the surface of Ca-zeolite was observed and it displayed a striking thrombin activation pattern, which exhibited an exceptionally high plateau thrombin activity and at least 12-fold enhanced endogenous thrombin compared to natural platelet-based physiological processes. Remarkably, various zeolite-based inorganic hemostatic materials have been marketed. QuikClot granular powder (QC) was approved for application by the FDA in 2002. For instance, Margulis *et al.* demonstrated that QC induced an immediate and continuous hemostasis.^118^ Similarly, Rhee *et al.* reported that QC controlled bleeding in 92% of cases for 103 military and civilian patients.^119^ However, QC had a serious side effect of causing tissue thermal damage. Wright and co-workers found that QC could cause an increased temperature at both the surface and in the interior of the tissue of about 95 °C and 50 °C.^120^ To deal with this challenge, Ostomel *et al.* modified a zeolite-based hemostatic agent by exchanging Ca^2+^ with Ag^+^. Hence, they greatly reduced the heat generation and endowed the hemostatic agent with an antibacterial ability.^121^ In another study, Yu and co-workers constructed an artificial catalyst composed of trypsin and a Ca^2+^-exchanged Y-type zeolite. This artificial catalyst exhibited strong hemostatic ability in animal models of massive hemorrhage and hemophilia without causing tissue thermal damage.^122^

#### 3.2.2. Kaolin

Kaolin is a hydrous aluminum silicate mineral, which exhibits a relatively low specific surface area, low cation exchange capacity and a minimal charge on the layer.^123,124^ There are several factors including the “glass effect” and negative surface charge that cumulatively contribute to the ability of kaolin in accelerating the body's natural blood clotting.^125^ Conventionally, the marketed kaolin-based inorganic hemostatic materials are mainly QuikClot Combat Gauze™ (QCG) approved by the FDA in 2013. QCG acts as a procoagulant, and when it comes in contact with blood, the kaolin immediately dissociates from the gauze and activates the intrinsic pathway. However, QCG is a non-absorbable hemostatic dressing that does not offer immediate hemostasis.^126^ For example, Gegel *et al.* assessed the efficacy of QCG *in vivo* and demonstrated that QCG could form a more robust thrombus to significantly reduce blood loss.^127^ Chavez-Delgado *et al.* further applied QCG to hemostasis of 230 patients undergoing tonsillectomy, and QCG achieved rapid and complete hemostasis in 84.8% of patients.^128^ Currently, inspired by a traditional Chinese medicine hematitum which consisted of iron oxide, Long and co-workers developed an emerging kaolin nanoclay composite (α-Fe_2_O_3_-kaolin_KAc_) through a facile precipitation method based on natural hemostatic agents. The results showed that the emerging kaolin nanoclay composite exhibited the properties of absorbing fluid, concentrating coagulation factors and activating the intrinsic coagulation pathway. Besides, α-Fe_2_O_3_ in this nanoclay composite could also promote blood cell aggregation and coagulation to further promote hemostasis.^129^

#### 3.2.3. Montmorillonite

Montmorillonite is also a hydrous aluminum silicate mineral, which possesses various properties, including a large specific surface area, cation exchange capacity and high viscosity due to its small particle size.^124,130^ When exposed to blood, the montmorillonite can absorb a large volume of water, thereby aggregating coagulation factors. Furthermore, the negative surface charge of montmorillonite also activates the intrinsic coagulation pathway.^130^ Montmorillonite has been commercialized as WoundStat™ (WS) approved by the FDA in 2007. WS can rapidly absorb water and swell into a clay paste with high plasticity and strong adhesiveness to form a physical barrier and stop bleeding. Nevertheless, WS is non-biodegradable and must be removed by physical means. What's more, residual WS particles in the body can easily lead to thromboembolism and complications. As an example, Ward *et al.* reported the application of WS in an arterial hemorrhage swine model, and histological examination revealed residual particles in the arteries, which might lead to thrombosis.^131^ In another study, Kheirabadi *et al.* evaluated the safety of WS. The results indicated that the majority of WS-treated vessels were occluded by intravascular thrombus formation, suggesting that WS might lead to thromboembolic risk.^132^ Although montmorillonite was reported to be an effective hemostatic agent to address massive bleeding, the inflammation and thrombosis led to restricted application.

### 3.3. Silica-based mesoporous particles

Recently, mesostructured materials such as mesoporous silica, mesoporous bioactive glasses, diatom silica and their composites have opened up a new direction in the field of hemostasis. Similar to natural mineral clays, silica-based mesoporous particles can rapidly absorb water and aggregate platelets, blood cells and coagulation factors at the injury site. On the other hand, the negative surface charge and released Ca^2+^ from silica-based mesoporous particles also activate the intrinsic pathway.^35^ Furthermore, silica-based mesoporous particles show much better biosafety than natural mineral clays, because silica or silicate processes excellent biocompatibility and degradability. Therefore, silica-based mesoporous particles displayed greater potential in hemostasis and have been extensively studied and applied.

#### 3.3.1. Mesoporous silica

Mesoporous silica (MS) has currently attracted worldwide attention because of its significant features including biocompatibility, tailorable surface charges, large pore sizes and pore volumes.^133,134^ For hemostasis, MS with a high specific surface area and porosity can promote hemostasis by absorbing a large amount of water and condensing coagulation factors.^135^ For example, Dai *et al.* developed a silver exchanged calcium doped ordered mesoporous silica sphere (AgCaMSS) for hemorrhage control. The AgCaMSS showed a pore size of 3.2 nm, BET surface area of 919 m^2^ g^−1^ and pore volume of 0.74 m^3^ g^−1^. *In vivo* experiments further demonstrated that the AgCaMSS could significantly promote blood clotting, activate the intrinsic pathway and induce platelet adherence.^136^ In another study, Wang and co-workers prepared tannic acid-loaded mesoporous silica nanoparticles (TMS). Hemostasis tests proved that the TMS could reduce the hemostatic time by 65% both *in vitro* and *in vivo*. TMS also exhibited antibacterial activity against *Staphylococcus aureus* and Staphylococcus epidermidis.^137^ Similarly, Bhat *et al.* developed new gated mesoporous silica nanoparticles (MSN) loaded with an anticoagulant drug. Moreover, the thrombin-dependent response was assessed and a significant increase in the coagulation time from 2.6 min to 5 min was found.^138^ Sun *et al.* aimed to improve the hemostatic activity of chitosan through incorporating MS and they eventually developed porous chitosan-silica composite microspheres (CSMS-S). Interestingly, CSMS-S exhibited abundant surface and inner macropores, which were important for rapid hemostasis. The whole blood clotting kinetics further showed that CSMS-S could form larger blood clots and reduce the hemostatic time from 114 s to 97 s.^139^

#### 3.3.2. Mesoporous bioactive glasses

A new generation of nanostructured bioceramics, referred to as mesoporous bioactive glasses (MBGs), was first developed by Yan *et al.* in 2004 *via* a sol–gel route process.^140^ It's worth noting that MBGs share a similar mesoporous structure to MS. Moreover, MBGs possess a large surface area, large pore volume and Ca^2+^ release for good hemostatic activity.^141,142^ Additionally, Ostomel *et al.* further investigated the hemostatic activity of MBGs. Encouragingly, they demonstrated that MBGs with a higher Si : Ca ratio (80% Si) possessed better pro-coagulation activity.^143^ Pourshahrestani and co-workers developed MBGs containing various concentrations of Ga_2_O_3_. The incorporation of a lower Ga_2_O_3_ content (1 mol%) into the MBG system improved structural properties including specific surface area, mesopore size and pore volume, which endowed MBGs with excellent hemostatic and antibacterial effects.^144^ Similarly, Mendonca *et al.* reported MBGs containing tantalum along with their potential application as hemostats. It was found that the Ta incorporation in MBGs caused a decreased surface area and pore volume. However, the Ta-MBGs still showed a significant hemostatic ability, revealing that Ta had a special hemostatic activity.^145^ In another research study, Zheng and co-workers combined MBGs and corn starch porous microspheres (CMSs) to prepare MBG@CMS particles. Notably, the MBG@CMS particles possessed a higher water absorption rate and activated both intrinsic and extrinsic coagulation pathways, thereby significantly shortening the blood clotting time.^146^

#### 3.3.3. Diatom silica

The recent advance in biotechnology has accelerated the discovery and development of new materials. For example, diatom silica is a precisely tuned nanostructured silica biomaterial with 3D porous structures.^147^ The diatom silica possesses various shapes, sizes, hierarchical pores, high porosity and specific surface area.^148^ Consequently, these features endow diatom silica with abilities of absorbing water, concentrating coagulation factors and promoting hemostasis.^149^ Lee *et al.* explored dramatic differences of special surface wettability between natural diatom silica and synthetic silica. The results showed that the surface properties of synthetic silica were hydrophobic and hemophobic, while diatom silica exhibited superhydrophilicity and even superhemophilicity. Apparently, such superhydrophilicity of diatom silica did not solely originate from nanoporous structures but from the synergy of high-density silanol anions and the nanoarchitecture, thus demonstrating the hemostasis potential of diatom silica.^150^ In another study, Luo *et al.* isolated the diatom *Navicula australoshetlandica* sp. to obtain diatom silica. Interestingly, this diatom silica had a specific “porous web” (6–8 nm) substructure in the ordered nanopores (165–350 nm) and they further exhibited a low hemolysis rate and shortened blood clotting time *in vivo*.^151^ Feng and co-workers developed a series of chitosan-coated diatoms (CS-diatoms) for hemorrhage control. As a result, the CS-diatoms prepared with 1% chitosan exhibited favorable biocompatibility, great fluid absorption and a desirable hemostasis effect.^152^ Likewise, Wang prepared chitosan/dopamine/diatom-silica composite beads (CDDs), and found that the porous internal structure of CDDs led to a large amount of water absorption, which contributed to rapid hemostasis.^153^

### 3.4. Inorganic–polymer composite hemostatic materials

In order to better take advantage of high hemostatic activities of inorganic hemostatic materials, researchers have prepared inorganic–polymer composite hemostatic materials by combining active inorganic components with polymers.^37,154–156^ The polymers mainly provide supporting structures and partial hemostatic activities, while the inorganic active components afford the major hemostatic and therapeutic functions. It is a common way to achieve a uniform and stable inorganic–polymer composite by combining active inorganic components and polymers. When applied to wounds, inorganic particles in the composites will not fall off or come into direct contact with the wound site to cause damage. Moreover, the gradual release properties of active inorganic components in the composites are beneficial to the wound healing process, which will not cause the sharp change of ion concentration and pH in the microenvironment. Therefore, inorganic–polymer composite biomaterials possess better biosafety and biocompatibility compared to powders. Additionally, the most obvious feature of inorganic–polymer composite hemostatic materials is their various material forms including sponges, membranes and hydrogels, endowing the composites with extensive applications.

#### 3.4.1. Sponge composites

**Fig. 4:**
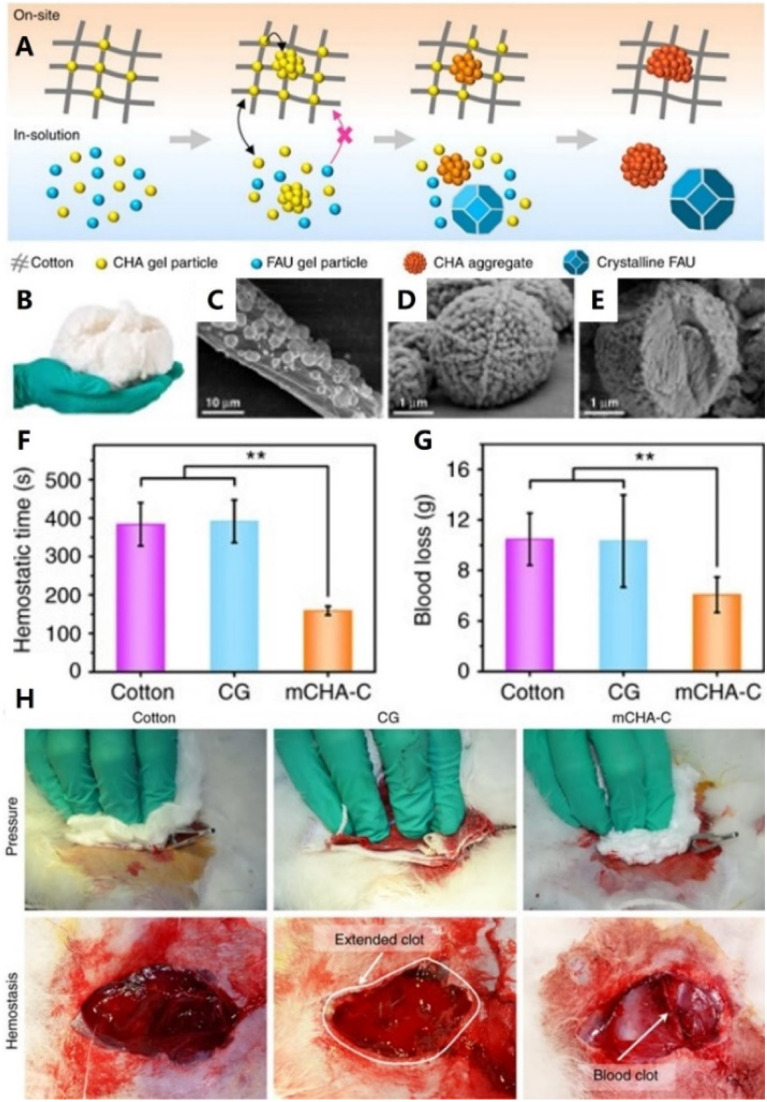
(A) Schematic representation of the tentative formation process mechanism of mCHA-C of on-site and in-solution products. (B) Photograph of mCHA-C. FE-SEM images of (C) mCHA-C, (D) mCHA zeolite on cotton, and (E) mCHA zeolite after removing cotton fiber by calcination. Quantitative analysis of hemostatic time (F) and blood loss (G) in a rabbit femoral artery injury model. (H) Hemostasis was assessed upon manual pressure on a rabbit lethal femoral artery injury with cotton, CG, or mCHA-C. Adapted with permission from ref. 156. Copyright 2019, CC BY 4.0.

As is known, sponge materials with high hydrophilicity and absorption capacity have been proven to be effective for promoting coagulation, owing to their ability of concentrating platelets, blood cells and coagulation factors. Conventionally, a sponge composite hemostat is fabricated through a specific molding technique (typically freeze-drying). Furthermore, these composite materials consisting of several active components can enable synergetic activation of the coagulation cascade and achieve better hemostasis. For instance, Jiang *et al.* used loose corn stalk and silver particles to adjust the solo chitin sponge. The prepared sponge showed a higher blood absorption ratio and lower blood clotting index.^37^ Pourshahrestani and co-workers constructed a series of 1% Ga_2_O_3_-containing mesoporous bioactive glass–chitosan composite sponges. The hydrophilicity and blood absorption rate of this sponge were significantly improved due to the high surface area of mesoporous bioactive glass. The results further revealed that the composite sponge exhibited increased capability to enhance thrombus generation and blood clotting.^154^ Similarly, Li *et al.* developed a hemostatic chitosan/diatom-biosilica-based aerogel. It's worth noting that the strong interface effect between dilation-biosilica and blood was favorable for promoting platelet aggregation and activating intrinsic pathway.^155^ In a recent study, Yu *et al.* tightly bound a mesoporous single-crystal zeolite onto the surface of cotton fibers to prepare a flexible zeolite-cotton hybrid hemostat (Fig. 4). On the one hand, zeolite particles were firmly anchored onto the cotton surface, thereby preventing tissue thermal damage. On the other hand, this hemostatic device had superior hemostatic performance over most other clay or zeolite-based inorganic hemostats.^156^ In another study, Zhang and co-workers synthesized mesoporous silica particles with large mesopores and fixed them on cotton fibers. Notably, this modified-cotton showed a shorter blood clotting time (86.00 s) and less blood loss (0.02 g) *in vivo*.^157^

#### 3.4.2 Membrane composite

Inspired by battlefield bandage, many inorganic–polymer composite membrane materials have also been developed. Different from sponge materials, the membrane hemostatic materials can be either hydrophilic^158^ or hydrophobic.^159^ On the one hand, the hydrophilic membranes can absorb water to aggregate coagulation factors. On the other hand, the hydrophobic membranes can act as a physical barrier to stop further bleeding. For example, Cui *et al.* reported a rapid effective nanoclay-based hemostatic membrane (NEM). The NEM with 60 wt% kaolinite (KEM) showed excellent hemostatic performance *in vitro* and *in vivo* benefiting from its enriched hemostatic functional site, robust fluffy framework, and hydrophilic surface. Furthermore, these results indicated that NEM acute hemostatic bandages were promising candidate materials for compressible hemorrhage control applications.^40^ Likewise, Delyanee M. and co-workers fabricated an electrospinning poly (lactic acid) and amino-modified halloysite nanotube (PLA/HNT) biomembrane. Interestingly, the negative surface charge of this biomembrane was greatly increased owing to the incorporation of HNTs and the intrinsic pathway of the coagulation cascade was activated faster. Hence, the blood clotting formation time decreased from 9 to 4 min.^160^ Chen *et al.* synthesized urushiol-functionalized mesoporous silica nanoparticles (MSN@U), which could form an amphipathic Janus membrane by interfacial self-assembly. The results showed that the Janus membrane possessed a large specific surface area, a rich porous structure and an ability of accelerating clotting cascade reactions.^161^ In another study, Jia *et al.* prepared a chitosan/mesoporous bioactive glass membrane for rapid hemostasis. The bioactive glasses with a mesoporous structure and high surface area endowed this membrane with better hydrophilicity and hemostatic activity. The results showed that the porous membrane possessed a good water absorption character and procoagulant activities in hemostasis.^162^

#### 3.4.3 Hydrogel composite

In addition to sponge and membrane composites, hydrogel-based hemostatic materials have also been extensively investigated and developed in recent years. In hemostasis, the hydrogel composite with good water absorption capacity can form a physical barrier at the wound site to stop bleeding after swelling and provide a moist ECM.^163–165^ For instance, Li *et al.* manufactured a maltose-like injectable hemostatic nanocomposite *via* combining chitosan polysaccharide and clay rectorite. Contributing to the hemostatic properties of rectorite, the viscous nanocomposite decreased the *in vitro* clotting time by 43%. Besides, an *in vivo* porcine skin model further confirmed the hemostatic performances of the viscous nanocomposite.^166^ Inspired by the great adhesive behavior of mussels and Arion subfuscus, Fan and co-workers prepared a novel polyacrylamide-tannic acid-kaolin hydrogel. Notably, the kaolin nanoparticles served as not only a physical crosslinking agent, but also an activator of the blood clotting factor FXII for accelerating coagulation.^167^ In another study, an injectable antibacterial conductive cryogel hemostat was reported by Zhao. This carbon nanotube incorporated cryogel displayed robust mechanical strength, rapid blood-triggered shape recovery and absorption speed together with high blood uptake capacity. Moreover, carbon nanotubes were discovered to be an activator of platelets and accelerate primary hemostasis.^168^ Mesoporous particles were also utilized to compound with polymer hydrogels. In two studies presented by Sundaram *et al.* and Shen *et al.*, MBG or MS and chitosan were composited into injectable hydrogels. Owing to the incorporation of mesoporous particles, these two kinds of hydrogels exhibited remarkably increased hemostatic effects.^169,170^

### 3.5 Self-supporting inorganic-based hemostatic materials

Generally, the content of active inorganic components in inorganic–polymer composites is relatively low (usually less than 30%),^37,156,167,171^ which motivated researchers to investigate hemostatic materials with both a high inorganic content and self-supporting structure. However, the major factor of materials to maintain the self-supporting structure is the interaction forces between the inorganic components (such as van der Waals forces, electrostatic interactions and intertwining). Hence, there are two main categories currently being studied—carbon materials and fiber materials.

#### 3.5.1 Carbon materials

Graphene and carbon nanotubes are two self-supporting inorganic carbon materials. As is known, graphene has been demonstrated to exhibit the function of activating platelets, thereby promoting blood coagulation and thrombosis formation.^172,173^ Graphene oxide (GO), a derivative of graphene, can form self-supporting sponges through electrostatic attraction and chemical bonding.^174^ Furthermore, graphene oxide possesses better hydrophilicity due to the oxygen-containing groups on its surface.^175^ For example, Liang *et al.* designed a zeolite/cross-linked graphene sponge (Z-CGS) which could control the heat release of the zeolite and maintain a stable low temperature. Thus, the Z-CGS sponge showed more significant hemostatic activities under the synergistic effects of thermal stimulation, electric charge stimulation and physical absorption capacity.^34^ Liang also developed a graphene-kaolin composite sponge (GKCS). Notably, the GKCS sponge could absorbed plasma and promoted the coagulation process without causing any harm such as cytotoxicity and hemolysis.^176^ Li and co-workers fixed montmorillonite (MMT) powder into cross-linked graphene sheets under a hydrothermal reaction and prepared a graphene-MMT composite sponge (GMCS). Encouragingly, the GMCS sponge with a 3D porous structure could absorb plasma, enrich coagulation factors and activate the coagulation cascade to achieve rapid hemostasis.^36^

Similar to graphene, carbon nanotubes (CNTs) have also been applied in hemostasis. CNTs show great advantages in hemorrhage control due to their excellent physicochemical properties including light weight, large specific surface area, high mechanical strength and platelet activation ability. From this perspective, Zhang *et al.* developed an effective three-dimensional hemostatic sponge (JWCNT/HBC) by chemical modification of a joint-welded carbon nanotube (JWCNT) sponge with hydroxybutyl chitosan (HBC). Encouragingly, the JWCNT/HBC sponge exhibited high elasticity, a porous structure, suitable blood-absorption and blood-maintaining performance.^177^

#### 3.5.2 Inorganic fibers

**Fig. 5:**
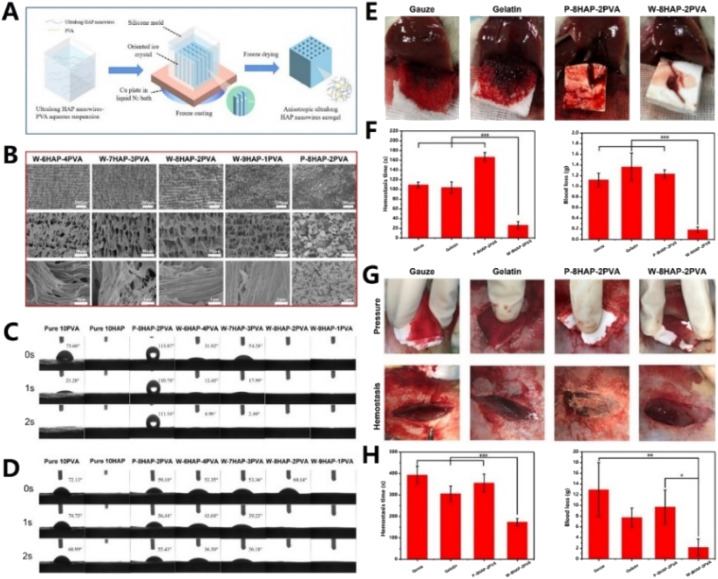
(A) Schematic illustration of the fabrication process of the W-HAP-PVA aerogel (composed of ultralong HAP nanowires and PVA). (B) SEM images of W-HAP-PVA aerogels with different compositions and P-8HAP-2PVA aerogel (consisting of 80 wt% HAP nanopowders and 20 wt% PVA). Comparison of the water contact angle (C) and blood contact angle (D) of W-HAP-PVA aerogels with different compositions, P-8HAP-2PVA, pure 10HAP and pure 10PVA aerogels. (E) Digital photos of hemostasis in rat liver puncture bleeding of W-8HAP-2PVA, P-8HAP-2PVA, gauze and gelatin sponge, and hemostatic capability of the materials: (F) hemostasis time and blood loss. (G) Digital photos of pressure and hemostasis in rabbit femoral artery injury bleeding of W-8HAP-2PVA, P-8HAP-2PVA, gauze and gelatin sponge. Hemostatic capability of the materials: (H) hemostasis time and blood loss. Adapted with permission from ref. 38. Copyright 2021, Elsevier B.V.

Another type of inorganic self-supporting material is inorganic fiber. Different from traditional inorganic powders with intrinsic rigidity and brittleness, inorganic fibers possess a high aspect ratio structure, flexibility and bendability.^178^ As we know, a variety of inorganic fibers (such as SiO_2_, Al_2_O_3_, and TiO_2_) have been used to prepare inorganic self-supporting sponges, aerogels and membranes. Furthermore, these inorganic self-supporting materials have been applied in the fields of thermal insulation, catalysis, oil–water separation, *etc.* However, there are still few studies about inorganic fiber materials for rapid hemostasis reported yet. Recently, our group fabricated an inorganic hemostatic aerogel mainly based on ultralong hydroxyapatite (HAP) nanowires (80% content) with polyvinyl alcohol (PVA) as the organic binder (as shown in Fig. 5). Interestingly, this HAP aerogel exhibited super-hydrophilicity and super-hematophilicity, which were favorable for promoting hemostasis. When applied in rat liver and rabbit femoral artery bleeding models, the hemostatic time and blood loss of this HAP aerogel were significantly decreased.^38^ Additionally, Li *et al.* fabricated biomimetic ‘‘cotton-like” hollow Al_2_O_3_ fibers (HFs) to address the challenges of hemostasis. The HFs possessed a hollow porous 3D structure, large specific surface area and negative Zeta potential. Moreover, the HFs could not only concentrate blood components and activate the intrinsic pathway, but also form HF-based clots with similar morphological characteristics to fibrin-based clots.^179^

## 4. Inorganic-based biomaterials for wound healing

In the process of wound healing, non-healing chronic wound or delayed wound healing always occurs due to various intrinsic and extrinsic factors.^180^ Autograft or allografts is considered as the most effective treatment option for wound healing, but it still suffers from inadequacies including limited donor source, risk of immune rejection and heavy financial burden.^181^ Recently, inorganic-based biomaterial are found to be promising candidates for wound healing.^182,183^ On the one hand, many inorganic materials with a small size can cooperate with polymers, resulting in forming hydrogels with skin-like structures.^184^ On the other hand, inorganic materials release many functional bioactive ions, which facilitate cell proliferation, migration or differentiation.^185^ Furthermore, the shelf availability and affordable price of inorganic materials will promote their development and application.^186^ Therefore, inorganic-based biomaterials hold great promise in acute and chronic wound healing.

### 4.1 Different composite forms of inorganic-based biomaterials for wound healing

As we know, inorganic biomaterials mainly exist in the form of particles and fibres.^187^ Considering that cutaneous damage is often large and varied in shape, the way of utilizing inorganic biomaterials alone for wound healing is limited.^188^ In order to meet the needs of different types of wounds, it is an important direction to incorporate inorganic biomaterials into other materials. Consequently, the composite materials are suitable for various wound types, greatly enhancing the application prospects of inorganic biomaterials.^189^

#### 4.1.1 Dressing

Generally, a wound dressing can be prepared by incorporating inorganic biomaterials into natural polymers and synthetic polymers.^190^ Natural polymer-based wound dressings have been extensively investigated including proteins (gelatin and collagen), polysaccharides (alginates and chitosan) and proteoglycans.^191^ Benefiting from controllable physical and chemical properties, synthetic polymers has also been used as wound dressings like poly(vinyl alcohol) (PVA), poly(ε-caprolactone) (PCL), and poly-(lactic acid) (PLA).^192^ However, the insufficient mechanical strength of natural materials and the poor biocompatibility of synthetic materials limit their development as wound dressings respectively.^193^ Inspiringly, the addition of inorganic biomaterials addressed these problems due to their favourable effect on stiffness, viscosity and bioactive environments.^194^ For example, Wang *et al.* designed a composite hydrogel dressing by adding CaO–SiO_2_–TiO_2_ (CST5) into alginate, and therefore the gelling ability and healing microenvironment were adjusted by using calcium and silicon ions respectively.^195^ Therefore, inorganic biomaterial-based wound dressings are one of the most widely used forms of wound healing materials.

Apparently, inorganic biomaterial-based wound dressings are quite suitable for continuous large wound defects owing to the characteristics of a small thickness, large area and full coverage for wounds.^196^ Correspondingly, the types of dressings mainly include hydrogel membranes, electrospinning films, and microneedle patches. Inorganic biomaterial-based hydrogels are widely implemented in wound dressings thanks to their soft, hydrophilic and biocompatible properties.^197^ Wang *et al.* prepared hydrogel membranes consisting of a series of black bioceramics with a chitosan matrix, which enhanced skin regenerative activity *in vivo*.^198^ In contrast, inorganic biomaterial-based electrospinning films have better flexibility and mechanical properties, serving as an important complement to wound dressings. Zhang *et al.* reported a kind of electrospinning film with Zn-doped hollow mesoporous silica nanospheres (HMZS) and polycaprolactone (PCL) that exhibited the ability to promote angiogenesis and wound healing.^199^ In recent years, inorganic biomaterial-based microneedle patches, a new category of innovative materials, have been increasingly brought into focus for wound dressings. They can penetrate through the epithelial basement membrane to access subcutaneous tissues and get a tighter fit with the wound, thereby preventing falling down and accelerating the healing rate.^200^

#### 4.1.2 Spray

In addition to continuous large wound defects, discontinuous wound defects or wounds with unfavourable topography are also common types of cutaneous damage. It has attracted great interest in clinical practice in recent years. Consequently, skin spray is utilized in the treatment of such wounds, owing to the advantages such as convenience of operation, adaptability to complex shapes and controllability of dressing thickness.^201^

Furthermore, inorganic biomaterial-based spray with therapeutic function has been greatly developed. Recently, our group developed a sprayable β-FeSi_2_-incorporated sodium alginate (FS/SA) hydrogel.^202^ It achieved timely healing of wounds with an irregular morphology induced by tumor. With the help of Fe ions and ˙OH generated under the tumor microenvironment, photothermal and chemodynamic therapies were allied to suppress tumors. Besides, favourable migration and differentiation of endothelial cells were induced by the bioactive Fe and Si ions in spray, contributing to the angiogenesis of skin wounds and efficient healing. In another study, Ouyang *et al.* designed a smart black phosphorus (BP)-based gel spray for diabetic ulcer treatment.^203^ On the one hand, such chronic wound healing was accelerated by promoting the proliferation of endothelial cells and vascularization. On the other hand, BP in the spray could response to NIR laser irradiation to generate local heat, thereby accelerating the microcirculatory blood flow and releasing the loaded drugs.

#### 4.1.3 Scaffold

Compared to dressing and spray, the scaffold has a porous structure that provides a favourable space and microenvironment to promote cell growth, adhesion and migration. Therefore, more and more 3D-printed scaffolds have been developed to repair injured skin.

**Fig. 6:**
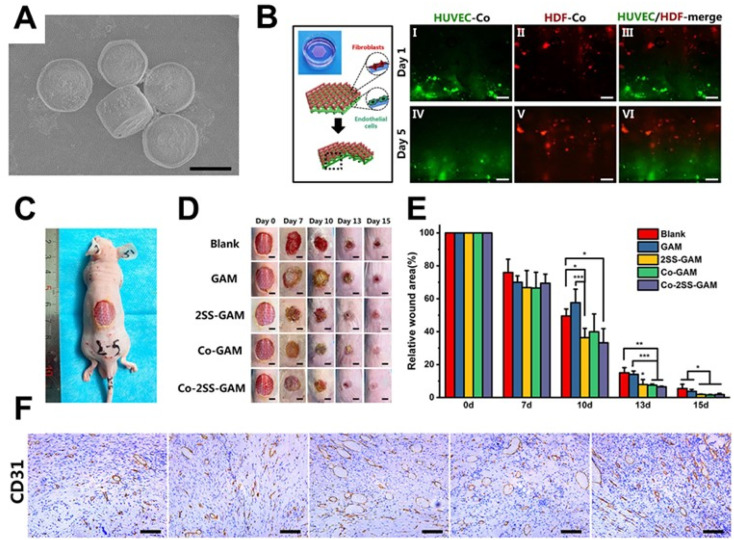
3D bioprinted scaffolds containing strontium silicate for wound healing. (A) SEM images of strontium silicate microparticles. (B) Schematic illustration and fluorescence images of the spatial distribution of HUVECs and HDFs in the scaffold. (C) Full-thickness skin defect model of nude mice. (D and E) Photos of wounds at different points of time and the corresponding statistics of wound closure rates. (F) Images of CD31 antibody immunohistochemical staining. Adapted with permission from ref. 204. Copyright 2021, Wiley-VCH GmbH.

Siebert *et al.* 3D printed a hydrogel scaffold encapsulating vascular endothelial growth factor (VEGF) decorated with tetrapodal zinc oxide (t-ZnO) microparticles. The scaffold had controlled the elastic modulus and degradation behavior by adjusting t-ZnO contents, and the *in vivo* wound healing was enhanced with the help of the scaffold.^205^ Considering the therapeutic effect of bioactive ions released from inorganic biomaterials, a CaSiO_3_-containing hydrogel scaffold was prepared by Ma *et al.* Such a hydrogel scaffold promoted the proliferation and migration of human umbilical vein endothelial cells and human dermal fibroblasts *in vitro* and enhanced angiogenesis and wound regeneration *in vivo*.^206^ However, for the regeneration of large-scale skin defects, a large number of cells are required, which is difficult to be satisfied through autologous cell proliferation. It is worth noting that cell-laden scaffolds are an emerging solution to this problem.^207^ Recently, our group synthesized strontium silicate (SS) microcylinders, based on which a novel multicellular scaffold was developed.^204^ On the one hand, the bilayered spatial distribution of vascular endothelial cells and fibroblasts in the scaffolds offered a vascularized skin-mimicking structure. On the other hand, the SS-based inorganic scaffold acted as a cell-induced factor to induce angiogenic vitality. Importantly, the positive effect of the bioprinted scaffolds on wound healing was verified in chronic wound and acute wound respectively (Fig. 6).

### 4.2 Therapeutic effects of inorganic-based biomaterials for wound healing

As is known, the process of wound healing is divided into four stages: hemostasis, inflammation, proliferation, and remodeling.^208^ Each stage has its own characteristics. For instance, in the inflammation stage, neutrophils infiltrate the injury site to kill bacteria and degrade damaged matrix proteins.^209^ Wound healing enters the proliferative stage 2–3 days after injury, in which the endothelial cells will across the basement membrane and further grow into capillaries or microvessels.^210^ Subsequently, some skin appendages such as hair follicles, sweat glands and sebaceous glands can regenerate during the remodelling stage.^211^ As a result, the development of inorganic-based biomaterials with specific therapeutic functions has become an effective way to promote wound healing.

#### 4.2.1 Antimicrobial properties

Metal oxides are common inorganic biomaterials with distinctive antibacterial performance, owing to their abilities to target cell wall, membrane, and cytoplasmic contents so as to disrupt cellular homeostasis.^212^ Various types of metal oxide-based composites have been developed in recent studies. Razali *et al.* prepared a titanium dioxide nanotube (TiO_2_-NT)-based bionanocomposite film.^213^ In this study, TiO_2_-NTs endowed the film with concentration-dependent antimicrobial properties, and the film containing 20% TiO_2_-NTs (w/w%) showed the best antibacterial performance, thereby significantly accelerating the wound healing rate in animal studies. Similarly, Ahmed *et al.* designed a ZnO-incorporated composite nanofiber membrane, and the antimicrobial properties conferred by ZnO nanoparticles promoted the wound healing in diabetic rabbits.^214^ Apart from metal oxides, silicate-based composites also exhibited antimicrobial properties during wound healing. For instance, CaCuSi_4_O_10_-containing nanofibrous membranes demonstrated that the Cu component could produce ROS to kill bacteria by destroying their cell walls or membranes.^215^ Likewise, another study reported a kind of wound dressing based on Cu-containing bioactive glass and eggshell membranes, in which a sustained release of Cu^2+^ ions distinctly inhibited the viability of bacteria.^216^

#### 4.2.2 Promoting angiogenesis

In normal tissues, the vasculature delivers adequate nutrients and oxygen to cells and remove carbon dioxide and waste products. Consequently, fluid accumulation, inflammation and hypoxia occur if angiogenesis is blocked during the wound healing.^217^ Therefore, developing inorganic biomaterials with angiogenesis performance is an attractive way of promoting wound healing. Bao *et al.* designed a four-layer composite bioglass-containing dressing by electrospinning and hot pressing. The results showed that the bioactive ions released from bioglass transferred to the wound bed for the stimulation of angiogenesis.^218^ As reported by Wu *et al.*, a bioscaffold with hollow manganese silicate nanospheres could enhance the expressions of angiogenic factors in endothelial cells by stimulating macrophages.^190^ The mechanism of inorganic-based biomaterials promoting angiogenesis has attracted more and more attention. For instance, Cu^2+^, Mg^2+^ and Sr^2+^ all promote vascular regeneration through activating eNOS and VEGF pathways.^219–221^

#### 4.2.3 Skin appendage remolding

Skin appendages, including hair follicles, sweat glands and sebaceous glands, play a vital role in the functions of normal tissue.^222^ Among them, hair follicles serve multiple functions including thermal insulation, physical protection, sensory perception, sebaceous dispersion and decorative purposes for social interactions, being the key to skin appendage remolding in wound healing.^223^ Excitedly, the studies of inorganic biomaterials for hair follicle regeneration have been under investigation for a long time. Zhang *et al.* synthesized Zn-doped hollow mesoporous silica nanospheres (HMZSs), which were incorporated into polycaprolactone (PCL) electrospun fibers to form a novel wound healing dressing. The Zn ions enhanced the recruitment and proliferation of hair follicle stem cells. Furthermore, Zhang *et al.* designed a biofluid-absorbing bioactive sandwich-structured Zn–Si bioceramic composite wound dressing. The results showed that the synergistic effect of the Zn^2+^/SiO_3_^2−^combination enhanced the gene expressions of hair follicle anagen markers including PDGF-A, PDGF-B, and C-Myc *in vitro*, and *in vivo* results further indicated that the synergistic activity activated the recruitment of hair follicle stem cells and promoted the regeneration of hair follicles.^224^ In addition, Zhang *et al.* also prepared a new type of composite composed of curcumin loaded Fe–SiO_2_ nanoparticles, and demonstrated that the synergistic effect of SiO_3_^2−^, Fe^3+^ and Fe-Cur chelates obviously inhibited scar hyperplasia and promoted hair follicle regeneration.^225^

### 4.3 Intelligently responsive inorganic-based biomaterials for wound healing

To date, the conventional wound dressings including bandages, hydrogels and foams have been widely explored.^226^ However, wound healing is a continuously changing dynamic process in which various chemical and physical changes take place. Therefore, external and internal stimuli such as light, pH, heat, and strain can influence the microenvironment of wound healing.^227–229^ Inorganic biomaterial-based wound dressings that can flexibly respond to these changes are one of the key therapeutants of the intelligent wound healing field.

#### 4.3.1 Light-responsive inorganic-based biomaterials for wound healing

Light, a particularly promising tool, holds the advantages of noninvasive nature, ease of application and spatiotemporal control. Effects derived from the reaction of inorganic biomaterials to light like photothermal therapy (PTT), photodynamic therapy (PDT), and a combination of PTT and PDT have been applied in the treatment of wound healing.^227^ Li *et al.* fabricated a NIR light-responsive hydrogel consisting of 3-(trimethoxysilyl) propyl methacrylate (MPS) and mesoporous silica (mSiO_2_) modified CuS nanoparticles for wound healing. The produced local heat under NIR light irradiation endowed the composite with controlled Cu^2+^ release behavior, leading to enhanced antibacterial efficacy and a faster wound healing rate.^230^ In addition to the antibacterial effect, the local heat generated by PTT plays an important role in tissue cells in wound healing as well. For example, Sheng *et al.* prepared a novel fayalite-based hydrogel with the photothermal effect and the heat stimulated angiogenesis through the VEGF and HSP90/eNOS signaling in endothelial cells. Subsequently, obvious enhancement of angiogenesis and chronic wound healing could be observed *in vivo*.^231^ PDT means generating (ROS) by using photosensitizers and appropriate excitation sources, thereby oxidatively damaging surrounding biomolecules and killing bacteria.^232^ Zhang *et al.* synthesized an efficient NIR-triggered antimicrobial photodynamic therapy system with lanthanide-doped upconversion nanoparticles (UCNPs, LiYF4: Yb/Er). The results demonstrated the high efficacy of the nanosystem for the inhibition of deep-tissue multi-drug resistant (MDR) bacteria *in vitro* and *in vivo*.^233^ However, single PTT or single PDT needs a higher temperature or abundant ROS respectively to achieve effective antibacterial properties, which may cause side effects to normal tissues.^227^ Therefore, the alternative strategy of employing synergistic photothermal/photodynamic therapy is practically needed for wound healing. Zhang *et al.* designed an effective antibacterial hydrogel embedded with Ag_3_PO_4_/MoS_2_ composites, which produced more ROS and more heat under dual light irradiation compared to PTT or PDT alone. As a result, the composite hydrogel exhibited excellent wound healing efficiency.

#### 4.3.2 pH-responsive inorganic-based biomaterials for wound healing

According to a previous study, the wound pH varies continuously during the different wound healing stages. A slightly acidic pH is beneficial to wound healing thanks to the increased fibroblast activity and limited bacterial proliferation.^234^ Hence, it is a good new idea for wound healing to design inorganic materials that respond to the wound pH and in turn act on the wound microenvironment. Recently, Wu *et al.* designed an injectable and pH-responsive nanocomposite hydrogel based on amine-modified silica nanoparticles. The results showed that the mechanical, drug release and hydrolytic degradation behaviours of this hydrogel could be adjusted in the mildly acidic range.^235^ Junior *et al.* successfully synthesized a series of poly (acid methacrylic)/LAPONITE® RDS nanocomposite hydrogels with different amounts of Lap RDS by free-radical polymerization. The variety of pH could adjust the hydrophilic properties of the nanocomposites, leading to a controlled release system in wound healing.^236^ However, these hydrogels have not been experimentally verified for the wound healing process. Excitedly, Guo *et al.* prepared a nanofibrous mat containing sodium bicarbonate by coaxial electrospinning, realizing pH-controlled dual-drug release according to the specific needs of various wound healing periods.^237^ It is believed that with the update of material preparation technology and the development of material properties, more and more pH-responsive inorganic materials will be applied in the treatment of cutaneous wounds.

#### 4.3.3 Other stimuli-responsive inorganic-based biomaterials for wound healing

In addition to the two most common stimuli mentioned above, some inorganic biomaterials that respond to other stimuli have also been developed for wound healing. Zhang *et al.* designed a novel wound dressing based on magnetothermally responsive microfibers composed of Fe_3_O_4_@SiO_2_. The smart constructs released antibiotics as needed to prevent infection. Subsequently, the controlled drug delivery significantly accelerates the healing process.^238^ Mao *et al.* developed a hydrogel composed of MXene (Ti_3_C_2_T_*x*_) which could electrically modulate cell behaviors. Benefiting from the unique conductivity of Ti_3_C_2_T_*x*_-MXene, the composite hydrogel actively enhanced the proliferation of NIH3T3 cells *in vitro* and significantly accelerated the wound healing rate *in vivo* under electrical stimulation.^239^ Mechano-responsive inorganic-based biomaterials are also a class of promising wound healing materials. Bhang *et al.* developed a zinc oxide nanorod-based piezoelectric patch, which generated electric potential difference-derived electrical fields (EFs) upon mechanical deformation caused by animal motion and induced piezoelectric potentials at the wound bed to facilitate wound healing. It was attributed to the promotion of cell migration, angiogenesis, and collagen synthesis under the EFs. With the concept of intelligent and refined wound management gradually recognized by the public, it is believed that stimuli-responsive inorganic materials will be gradually translated into the clinic and become the next generation of efficient wound repair tools.

In recent years, most of studies on inorganic-based biomaterials for wound healing have been focused on skin regeneration, because the skin is the body's largest organ and vulnerable to injury due to direct contact with the environment. However, there are also some typical examples for healing other soft tissues like cornea, oral mucosal or muscle with inorganic-based biomaterials. For instance, Wu *et al.* developed a wireless-powered electrical bandage contact lens (EBCL) by depositing a Cu film on a flexible polyimide substrate. This equipment generated an external electric field *in situ* to stimulate the corneal epithelial cells, thereby accelerating the corneal wound healing.^240^ Chen *et al.* prepared a porous polydroxyalkanoate (PHA) scaffold containing zinc oxide nanoparticles (ZnO NPs) for oral soft tissue regeneration. The presence of ZnO NPs resulted in the release of ROS to prevent bacterial growth. Subsequently, the rat oral soft tissue defect after infection was repaired better under the treatment of the scaffold.^241^ Similarly, Elshazly *et al.* utilized bioactive glass nanofibers (BGnf) of composition B_2_O_3_, SiO_2_, and CaO to enhance oral mucosal wound regeneration in diabetes mellitus.^242^ Interestingly, Zhang *et al.* designed injectable conductive microcryogels based on reduced graphene oxide (rGO) and gelatin (GT). The addition of rGO significantly improved myogenic proliferation, differentiation and *in situ* muscle regeneration.^243^ All in all, inorganic-based biomaterials have been widely developed and used for wound healing, no matter whether for skin or other soft tissues.

## 5. Inorganic-based biomaterials for bifunctional rapid hemostasis and wound healing

As mentioned above, wound healing is a complex multiphase physiological process that includes four overlapping phases. On the one hand, most of the inorganic-based biomaterials for hemostasis only act in the hemostasis phase, that is, to stop bleeding in the early stage of wound formation. When bleeding stops, the inorganic-based biomaterial for hemostasis is removed along with adhering blood clots and does not participate in the subsequent phases of wound healing. On the other hand, inorganic-based biomaterials for wound healing mainly act in the phases of inflammation, proliferation and remodeling. They are fixed and maintained at the wound site to provide the necessary moist environment and bioactive ions over a period of time. Hence, there is almost no connection between single-functional inorganic-based biomaterials for hemostasis and single-functional inorganic-based biomaterials for wound healing because they act in different phases of wound healing. It had led to a bottleneck in the application of inorganic-based biomaterials, because single functional biomaterials were increasingly difficult to meet the complex and diverse clinical needs. In recent years, there has been increasing evidence that hemostasis is closely related to wound healing,^46,48,244–246^ thereby further driving research interest in the design of inorganic-based biomaterials for both rapid hemostasis and wound healing. For instance, blood clots are the final structure formed during the hemostatic phase and were composed mainly of platelets and fibrin, and numerous studies have shown that blood clots play decisive roles in promoting wound healing.^247^ Furthermore, blood clots not only play the role of fixing and connecting nearby tissues, but also provide a good immune microenvironment for the invasion of inflammatory cells and the migration and proliferation of fibroblasts.^248^ Therefore, if blood clots formed after hemostasis are stably fixed and maintained, the wound healing process can be better promoted. Inorganic-based biomaterials for bifunctional rapid hemostasis and wound healing can be achieved through the following aspects. One is that inorganic-based biomaterials exhibit dual functions of rapid hemostasis and wound healing, and these inorganic-based biomaterials can be applied for either the rapid hemostasis stage or the wound healing stage. Another is that inorganic-based biomaterials possess the ability of maintaining clots, which means that biomaterials can absorb blood, form clots, and play a role along with blood clots in the wound healing process without being removed or peeled off. For example, inorganic-based biomaterials that not only promote blood coagulation but also exhibit functions such as antibacterial and bioactive ion release to accelerate wound healing are the former category. Meanwhile inorganic-based biomaterials that quickly absorb blood to form blood clots and gradually degrade at the wound site to provide the necessary microenvironment and active ingredients for wound healing are the latter category.

### 5.1 Dual functional inorganic-based biomaterials

Many studies have reported the preparation and development of inorganic-based biomaterials with dual functions of rapid hemostasis and wound healing. From this perspective, silver nanoparticles (AgNPs) and their derivatives with both hemostatic and antibacterial activities are the most commonly used active inorganic components, and they can act in the hemostasis phase and inflammation phase (reducing inflammation by suppressing bacterial infections) to achieve better therapeutic effects. For example, Wu *et al.* used thiol-modified chitosan and AgNPs to prepare a composite sponge (TMC/AgNPs) for stemming the bleeding and preventing infection. The TMC/AgNP sponge had a complex interlaced tubular porous structure with high porosity (99.42%). Moreover, the TMC/AgNP sponge exhibited excellent antibacterial activity against *Staphylococcus aureus*, *Escherichia coli*, and *Pseudomonas aeruginosa*. *In vivo* experiments further confirmed its efficient hemostatic and wound healing performances.^249^ Similarly, Lan and co-workers designed a chitosan hemostatic dressing with AgNPs (CGSH/Ag_50_), and found that CGSH/Ag_50_ could promote platelet aggregation by the activation of the positively charged surface and stimulation of AgNPs. Besides, this dressing showed high liquid absorption capacity, good hemostatic activity and antibacterial activity *in vivo*, thereby significantly accelerating full-thickness wound healing.^86^ To overcome the challenges in controlling the size and colloidal stability of AgNPs, Shakya *et al.* developed a template-guided synthesis of ultrafine AgNPs around 2 nm using water-soluble and biocompatible γ-cyclodextrin metal–organic frameworks (CD-MOFs). The ultrafine AgNPs could be easily dispersed in aqueous media and exhibit effective bacterial inhibition. Furthermore, the synthesized AgNPs@CD-MOFs showed a boosted hemostatic effect that further enhanced wound healing in synergy with the antibacterial effect.^250^ In another study, Zhang *et al.* reported a green strategy for *in situ* biomimetic syntheses of silver nanoparticles@organic frameworks/graphene oxide in a sericin/chitosan/polyvinyl alcohol hydrogel. Contributing to AgNPs, this composite dressing showed excellent lasting antibacterial properties against drug-sensitive and drug-resistant pathogenic bacteria. Interestingly, the hydrogel possessed a coagulation effect because it aggregated and acted on blood cells and platelets. *In vivo* evaluation further demonstrated that the composite dressing could achieve rapid hemostasis, and accelerate wound healing and re-epithelialization.^251^ Xu and co-workers demonstrated a multifunctional cryogel to block acute hemorrhage and promote wound healing, and this composite cryogel was composed of chitosan, silver and tannic acid (CS/Ag/TC). Due to the porous structure and positive charge of CS, the prepared cryogel exhibited good hemostatic capability with a hemostasis time less than 20 s. Besides, the CS/Ag/TC cryogel showed good antibacterial activity and effective oxidation resistance attributed to Ag and TA molecules. Under the synergetic effects of hemostasis, antibacterial activity and oxidation resistance, this cryogel could significantly promote wound repair in the skin incision model.^252^

In addition to AgNPs, other active inorganic components have also been investigated to fabricate composite biomaterials for both rapid hemostasis and wound healing. For instance, Ren *et al.* successfully prepared a kind of nanofiber dressing blended with silk fibroin, chitosan and drug-loaded halloysite nanotubes (HNTs). The addition of HNTs had a significant effect on the hemostatic activity of nanofibers and led to rapid coagulation of blood *in vitro*. Furthermore, this medical dressing presented stable antibacterial activity for wound healing owing to its drug release ability.^254^ To enhance the therapeutic effects of chitosan, Zhou and co-workers simply doped CaCO_3_ into acetate chitosan to form a wound dressing. Interestingly, the H^+^ reacted with CaCO_3_ to produce a mass of Ca^2+^ after absorbing water, resulting in activating secondary hemostasis. The released Ca^2+^ and residual CaCO_3_ further cross-linked with chitosan to form a tough and adhesive hydrogel to prevent further bleeding. Additionally, the wound dressing exhibited excellent hemostatic efficacy and accelerated wound healing through promoting re-epithelization and collagen deposition *in vivo*.^255^ In another study, Rao *et al.* synthesized a hemostatic and antibacterial biomaterial, namely a ZnO nanocomposite using mushroom carboxymethyl chitosan as a natural polymer stabilizing agent. As a result, the ZnO nanocomposite showed enhanced antibacterial activity toward *S. aureus* ascribed to the synergetic activities of mushroom carboxymethyl chitosan and ZnO. Moreover, this nanocomposite had acceptable hemostatic properties for use in wound dressing and cosmetic applications.^256^ Inspired by the mechanisms of blood clot formation and secondary coagulation cascade activation in the natural hemostasis process, Wang *et al.* prepared an injectable hydrogel sponge (QHM) consisting of hydroxyethyl cellulose and mesocellular silica foam. The QHM exhibited instant water-triggered expansion and superabsorbent capacity, thereby synergistically promoting hemostasis. Moreover, the remarkable antibacterial activity and excellent cytocompatibility of the QHM were also observed *in vitro*. With efficient hemostatic efficacy and excellent antibacterial behavior, the QHM dramatically facilitated the wound healing in a full-thickness skin defect model.^53^

### 5.2 Clot maintaining inorganic-based biomaterials

**Fig. 7:**
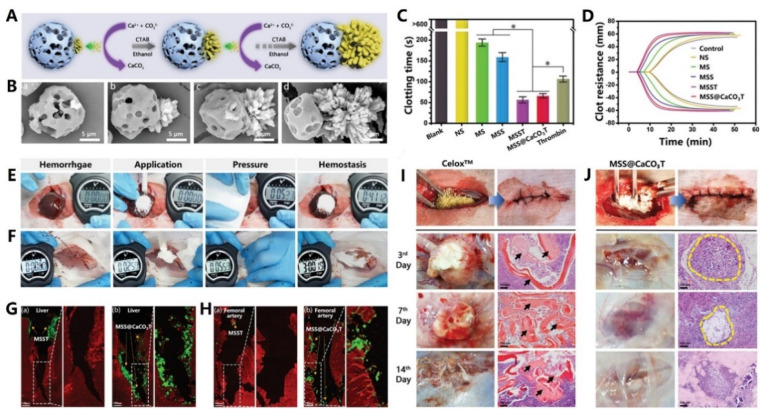
(A) Schematic showing the growth of flower-like CaCO_3_ crystals on negative-potential-charged microporous starch (MSS) during the synthesis of Janus MSS@CaCO_3_ particles. (B) SEM images of MSS@CaCO_3_ Janus particles with different aspect ratios of [MSS]/[CaCO_3_]: (a) 1 : 0.1, (b) 1 : 0.5, (c) 1 : 1, and (d) 1 : 2. (C) Whole blood *in vitro* clotting time. (D) Representative TEG parameters of whole blood treated with particles. Images of hemostasis in liver (E) and femoral artery (F) bleeding models. Histological sections from (G) liver bleeding models and (H) femoral artery bleeding models treated with (a) MSST and (b) Janus MSS@CaCO_3_T. Histological sections from *in vivo* biodegradation models treated with Celox (I) and Janus MSS@CaCO_3_T (J). Adapted with permission from ref. ^253^. Copyright 2020, Wiley-VCH GmbH.

Considering the key role of blood clots in the wound healing process, utilizing inorganic-based biomaterials for fixing blood clots and acting in the wound site still remains a challenge. From this perspective, it requires inorganic-based biomaterials to possess good tissue adhesion, excellent biocompatibility and hemostatic activity, so as to stably fix blood clots. Furthermore, inorganic-based biomaterials should slowly degrade and release bioactive ions at the wound site to promote wound healing. For example, Nie *et al.* prepared silver nanoparticle-incorporated mesoporous silica granules (AgNP-MSG) for hemostasis and repair of internal organs. *In vitro* experiments demonstrated that the as-prepared composite showed high absorption capacity, hemostasis efficacy, good biocompatibility and sustained antibacterial activity. Besides, AgNP-MSG was applied in a rat liver injury model for hemorrhage control and organ healing. Encouragingly, bleeding was effectively controlled within 7 s and AgNP-MSG was retained with blood clots in the injured liver. After two weeks, the rat survived and the liver was almost fully repaired with no trace of AgNP-MSG being found.^257^ In another study, Li *et al.* developed biphasic Janus self-propelled hemostatic particles loaded with thrombin (MSS@CaCO_3_T). Notably, these Janus particles were implanted in a rabbit back subcutaneous muscle injury model after bleeding. Contributing to the release of thrombin and Ca^2+^, MSS@CaCO_3_T could significantly accelerate blood clotting *in vivo* for activating the coagulation cascade. After the implantation of Janus MSS@CaCO_3_T for 14 days, histological analysis further confirmed that MSS@CaCO_3_T particles could gradually degrade and promote tissue repair. Hence, MSS@CaCO_3_T particles with excellent hemostatic efficacy and biodegradation displayed great potential in the therapy of complex traumas (Fig. 7).^253^ To achieve rapid, strong hemostasis as well as closure and healing in deep wounds, Meddahi-Pelle and co-workers prepared aqueous solutions of Stober silica or iron oxide nanoparticles that exhibited nanobridging function. It was shown that aqueous solutions of nanoparticles could stop bleeding within a minute in a rat liver organ injury and promote healing of the organ injury due to its instant hemostasis, tight closure and rapid degradation.^258^ Currently, Liu *et al.* developed a portable electrospinning device (150 g in weight) for outdoor hemostasis and further utilized this device to green-synthesize CuS composite nanofibers. Interestingly, the CuS composite nanofibers could be deposited *in situ* and possessed better compactness onto the rough wound surface than conventional nanofiber mats. Furthermore, they are favorable to simultaneously achieve rapid hemostasis outdoors and ablate superbacteria, resulting in promoting wound healing. Thus, the CuS composite nanofibers not only accelerate the blood coagulation (<6 s) but also shorten the healing time of the wound with superbacterial infection (18 days).^29^ Although many inorganic-based biomaterials for bifunctional rapid hemostasis and wound healing have been successfully prepared, some current bottlenecks and problems need to be addressed. Firstly, components and forms of inorganic-based biomaterials for bifunctional rapid hemostasis and wound healing are mostly powders, represented by AgNPs. However, powders for wounds could lead to a sharp release of ions and a large change in pH, which were negative for tissue healing. Moreover, residual particles in the body might increase the risk of thromboembolism. Combining active inorganic components with polymers is a development trend to address this problem. Secondly, most inorganic-based biomaterials for bifunctional rapid hemostasis and wound healing only integrate hemostatic and antimicrobial effects. Nevertheless, the process of wound healing also needs the avoidance of excessive inflammatory response, a suitable immune microenvironment and a large number of extra tissue cells. It means that inorganic-based biomaterials are required to combine hemostasis with more functions including anti-inflammation, immunoregulation, promotion of cell proliferation and differentiation, and tissue remolding. It is still a challenge to connect different phases of wound healing with inorganic-based biomaterials and have a cascade effect to achieve better therapeutic outcomes. Hence, it is important to develop more inorganic-based biomaterials with multifunctional properties, which requires scientists to further carry out more innovations and attempts.

As discussed above, there are various inorganic-based biomaterials with different biofunctions that have been extensively developed. In order to facilitate the reading and understanding for readers, we have sorted out and summarized the above contents. Table 1 lists some representative types of inorganic-based biomaterials that can be applied to rapid hemostasis and wound healing.

**Table 1:** Inorganic-based biomaterials for rapid hemostasis and wound healing

Application field	Material type	Active inorganic component	Biofunctions	Ref.
Rapid hemostasis	Natural mineral clays	Zeolite, kaolin, montmorillonite	Blood absorption, Ca^2+^ release, negative surface charge, mechanical strength enhancement, tissue adhesive	121 and 128
	Silica-based mesoporous particles	AgCaMSS, CSMS-S, Ga_2_O_3_-MBGs, Ta-MBGs, diatom silica	Blood absorption, high surface aera and pore volume, Ca^2+^ release, negative surface charge	135, 138, 143 and 150
	Inorganic–polymer composites	Mesoporous single-crystal zeolite, NEM, HNTs, rectorite	Blood absorption, concentrating coagulation factors, negative surface charge, tissue adhesive, physical barrier	40, 155, 159 and 165
	Self-supporting inorganic-based biomaterials	Graphene, GO, CNTs, ultralong HAP nanowires, hollow Al_2_O_3_ fibers	Blood absorption, super hydrophilicity and hemophilicity, high surface aera, activation of platelets	36, 38, 150–153, 176 and 178
Wound healing	Basic dressings	CST5, FS, BP, t-ZnO, CaSiO_3_, SS	Bioactive ion release, angiogenesis, PTT, CDT, skin defects regeneration	194, 201, 203 and 205
	Therapeutic functional inorganic-based biomaterials	TiO_2_-NTs, CaCuSi_4_O_10_, HMZS, hollow manganese silicate nanospheres	Bioactive ion release, antibacterial activity, angiogenesis, skin appendage remolding, hair follicle regeneration	38, 179, 189, 212 and 215
	Intelligently responsive inorganic-based biomaterials	Ag_3_PO_4_/MoS_2_, LAPONITE® RDS, Ti_3_C_2_T_*x*_, Fe_3_O_4_@SiO_2_, MoS_2_ nanosheet	Antibacterial activity, angiogenesis, pH-responsive, PTT, PDT, controlled drug delivery, electrical stimulation	232–236
Rapid hemostasis and wound healing	Dual functional inorganic-based biomaterials	AgNPs, HNTs, CaCO_3_, ZnO mesocellular silica foam	Blood absorption, negative surface charge, hemostasis drug release, antibacterial activity, reduce inflammation, promote re-epithelization and collagen deposition	53, 243, 245 and 246
	Clot maintaining inorganic-based biomaterials	AgNP-MSG, MSS@CaCO_3_T, CuS, Stober silica and iron oxide	Blood absorption, negative surface charge, tissue adhesive, clot maintaining, antibacterial activity, degradability, bioactive ion release, biocompatibility	29, 248–250

## 6. Conclusions and prospects

Inorganic-based biomaterials for rapid hemostasis and wound healing are essential in wound treatment, especially in the cases of severe bleeding when the inherent hemostatic system fails to stop the bleeding. In this article, we systematically review inorganic-based biomaterials for hemostasis and wound healing from different perspectives. Components and material forms are two key factors to determine the properties and functions of inorganic-based biomaterials. The components with hemostasis and wound healing functions have been well studied over the past 20 years, leading to the identification of new active inorganic components. Meanwhile, researchers provide deep insight into their mechanisms of stopping bleeding and promoting healing. The forms of materials also affect hemostasis and healing processes, and various forms of inorganic-based biomaterials have been developed. However, each form of inorganic-based biomaterials has its own limitations, which require additional investigations to identify innovation solutions. Hence, it is an important issue to discuss the challenges and prospects in the material design, functions and applications of inorganic-based biomaterials for rapid hemostasis and wound healing.

Material design determines the composition and structure of biomaterials. However, most of current design routes to inorganic-based biomaterials are complicated and time-consuming. The principles of green chemistry and the cost of the ingredients should also be considered before developing a new synthetic method. It is worth noting that inorganic-based biomaterials prepared in recent years mostly lack pertinence. This means that researchers need to pay more attention to the intrinsic mechanisms of hemostasis and wound healing, thereby designing targeted biomaterials. For instance, a potential research direction is to design inorganic-based biomaterials that participate in or promote physiological hemostasis and wound healing processes, and this strategy has not yet been widely targeted in existing studies. The design routes in current studies mainly depend on the final performances of inorganic-based biomaterials, while ignoring how the biomaterials participate in physiological hemostasis and wound healing processes. Hence, designing inorganic-based biomaterials to mimic native components and processes of hemostasis and wound healing is an appealing future trend.^259,260^ For example, the design of smart biomaterials such as platelet-like particles and fibrin-structured nanofibers could provide great exciting opportunities in the development of inorganic-based biomaterials. Although numerous types of inorganic-based biomaterials for rapid hemostasis and wound healing have been developed, only a few of them have reached the clinical trial stage of development. Nowadays, only several inorganic-based biomaterials containing natural mineral clays^261^ or silica-based mesoporous particles^262^ have been applied in clinical application, because they have easy-to-use material forms and active components approved by regulatory authorities. In general, inorganic-based biomaterials comprising simple chemicals, components and material forms are more likely to have access to clinical trials.

Although inorganic-based biomaterials with a single function have been widely studied, they are unable to meet the increasing clinical needs. Multifunctional inorganic-based biomaterials that integrate hemostatic ability with one or more other characteristics or functions are particularly desirable for clinic applications. As discussed above, inorganic-based biomaterials with multiple functions including hemostasis, antibacterial, immunoregulation, angiogenesis, *etc.* show great potential for wound treatment. Such situations have attracted more attention and more novel inorganic-based biomaterials are expected to be fabricated for better clinical therapeutic effects. The wound healing process is complicated and most of the inorganic-based therapeutic biomaterials only focus on skin repair. Unfortunately, the biomaterials that can promote the repair of internal organs are very rare although they belong to an important area that requires increased research attention.^263^ Besides, inorganic-based biomaterials with diagnostic and monitoring abilities have advantages over traditional materials. The pH, temperature and electrical properties of a wound site always change during hemostasis and wound healing processes. Therefore, it is highly promising to develop materials with the ability of monitoring these physiochemical changes at the wound sites so as to determine wound's conditions without peeling off the dressing. It also enables doctors and patients to better track the wound healing process. Another challenge is to design inorganic-based biomaterials that are easy to remove without peeling off blood clots and causing secondary bleeding or biomaterials that can be maintained with blood clots in the wound and gradually degrade. Hence, the biocompatibility of degradation biomaterials and the risk of an *in vivo* thrombo-inflammatory response should be taken into consideration. Determining how to control the degradation of inorganic-based biomaterials is another important issue, as the degradation rate should match the tissue repair process and the degradation products should be absorbed by around histiocytes.

The ultimate goal of biomaterials is to be applied in clinical practice for reliable therapeutic effects. It means that inorganic-based biomaterials for rapid hemostasis and wound healing should not only exhibit good therapeutic function, but also show easy operation in practical applications. However, most research in the lab rarely focuses on the application of biomaterials, which is not conducive to their clinical trials and industrialization. There are various practical properties including long-term stability and portability that need to be considered in developing inorganic-based biomaterials for rapid hemostasis and wound healing. For example, portable inorganic-based biomaterials that can be stored for a long time even under extreme conditions are particularly important for military and first-aid applications. The cost of inorganic-based biomaterials is also a key indicator, determining whether they can be mass-produced and developed for civilian use. Furthermore, many inorganic-based biomaterials for rapid hemostasis and wound healing are applied externally, so their aesthetics should be carefully considered.

In summary, further studies on inorganic-based biomaterials for rapid hemostasis and wound healing need to focus on more than effective hemorrhage control and wound healing. Numerous studies reported various inorganic-based biomaterials with different components, forms, properties or functions, and they showed excellent therapeutic activities in rapid hemostasis and wound healing. Nevertheless, there are still some obvious deficiencies, such as complicated synthesis and fabrication processes (time-consuming and costly) and a narrow application field (mostly in skin wounds). It is believed that the advancement of chemistry design and fabrication technology will greatly promote the development of inorganic-based biomaterials with hemostasis and wound healing properties, thereby accelerating their clinical application in the near future.

## Author contributions

C. Wu and Y. Zhu supervised the project and reviewed and revised the manuscript. Y. Zheng and J. Wu co-wrote the manuscript.

## Conflicts of interest

There are no conflicts to declare.

## Supplementary Material
